# Transcranial direct current stimulation (tDCS) in elderly with mild cognitive impairment: A pilot study

**DOI:** 10.1590/1980-57642018dn13-020007

**Published:** 2019

**Authors:** Marcos Alvinair Gomes, Henrique Teruo Akiba, July Silveira Gomes, Alisson Paulino Trevizol, Acioly Luiz Tavares de Lacerda, Álvaro Machado Dias

**Affiliations:** 1 Department of Psychiatry, Federal University of São Paulo, SP, Brazil.; 2 Department of Morphology, Faculty of Medical Sciences at São Paulo´s Holy House, São Paulo, SP, Brazil.

**Keywords:** mild cognitive impairment, elderly, tDCS, memory improvement, comprometimento cognitivo leve, idosos, ETCC, melhora da memória

## Abstract

Transcranial direct current stimulation (tDCS) is a non-invasive, painless and easy-to use-technology. It can be used in depression, schizophrenia and other neurological disorders. There are no studies about longer usage protocols regarding the ideal duration and weekly frequency of tDCS.

**Objective:**

to study the use of tDCS twice a week for longer periods to improve memory in elderly with MCI.

**Methods:**

a randomized double-blind controlled trial of anodal tDCS on cognition of 58 elderly aged over 60 years was conducted. A current of 2.0 mA was applied for 30 minutes for 10 sessions, twice a week. The anode was placed over the left dorsolateral prefrontal cortex (LDLFC). Subjects were evaluated before and after 10 sessions by the following tests: CAMCOG, Mini-Mental State Examination (MMSE), Trail Making, Semantic Verbal Fluency (Animals), Boston naming, Clock Drawing Test, Word list memory (WLMT), Direct and Indirect Digit Order (WAIS-III and WMS-III) and N-back.

**Results:**

After 10 sessions of tDCS, significant group-time interactions were found for the CAMCOG - executive functioning (χ^2^ = 3.961, p = 0.047), CAMCOG - verbal fluency (χ^2^ = 3.869, p = 0.049), CAMCOG - Memory recall (χ^2^ = 9.749, p = 0.004), and WMLT - recall (χ^2^ = 7.254, p = 0.007). A decline in performance on the CAMCOG - constructional praxis (χ^2^ = 4.371, p = 0.037) was found in the tDCS group after intervention. No significant differences were observed between the tDCS and Sham groups for any other tasks.

**Conclusion:**

tDCS at 2 mA for 30 min twice a week over 5 consecutive weeks proved superior to placebo (Sham) for improving memory recall, verbal fluency and executive functioning in elderly with MCI.

## RESUMO.

A ETCC (estimulação transcraniana por corrente contínua) é uma tecnologia não-invasiva, indolor e de fácil utilização. Pode ser usada na depressão, esquizofrenia e outros distúrbios neurológicos. Não há orientações ideais sobre o uso de protocolos mais longos quanto à duração e frequência semanal da ETCC.

**Objetivo:**

estudar o uso de ETCC duas vezes por semana por 5 semanas em idosos com CCL.

**Métodos:**

o estudo foi controlado, randomizado, duplo-cego com ETCC anódica em 58 idosos acima de 60 anos. Uma corrente de 2,0 mA foi aplicada por 30 minutos durante 10 sessões consecutivas, 2 vezes por semana. O ânodo foi colocado no córtex pré-frontal dorsolateral esquerdo (LDLFC). Os pacientes foram avaliados antes e após 10 sessões pelos testes: CAMCOG, Mini-Exame do Estado Mental (MMSE), Trilhas, Fluência Verbal Semântica - Animais, Boston, Relógio, Memória da Lista de Palavras (WLMT), Dígitos - ordem direta e indireta (WAIS-III e WMS-III) e N-back.

**Resultados:**

foram encontradas interações significativas (tempo/grupo) para CAMCOG - funcionamento executivo (χ^2^ = 3,961, p = 0,047), CAMCOG - fluência verbal (χ^2^ = 3,869, p = 0,049), CAMCOG - recuperação da memória (χ^2^ = 9.749, p = 0,004), WMLT - recordação (χ^2^ = 7,254, p = 0,007). Foi observado um declínio no grupo ETCC após a intervenção para CAMCOG - praxia construtiva (χ^2^ = 4,371, p = 0,037). Não encontramos diferenças significativas entre os grupos ETCC e placebo para outros testes.

**Conclusão:**

A ETCC de 2 mA por 30 min, 2x por semana, por 5 semanas consecutivas, é superior ao placebo (Sham) na melhoria da recuperação de memória, fluência verbal e funcionamento executivo em idosos com CCL.

Transcranial Direct Current Stimulation (tDCS) is associated with cognitive improvements in healthy individuals,^1^^,^ 2 modulating cortical excitability through synaptic long-term potentiation/depression rate.^3^ The most important objective of tDCS is to modulate neuronal activity of some specific brain areas in a polarity-dependent pathway.^4^ During stimulation, current flows into the brain between the electrodes, modulating the brain such that the region beneath the anode undergoes depolarization resulting in excitation, while the area beneath the cathode undergoes hyperpolarization and inhibition.^5^ Although many authors have studied the effects of tDCS for mental disorders,^6^ there is no clear consensus on applying this technique in dementia-related disorders.^7^ Mild Cognitive Impairment (MCI) may represent a prodromal stage of Alzheimer’s dementia.^8^ Many studies have suggested a progression rate of MCI to dementia averaging around 10% to 15% per year, particularly in amnestic MCI, where executive cognition disabilities are prevalent.^9^

In the complex physiopathology of MCI, many authors describe a dorsolateral prefrontal cortex (DLPFC) dysfunction. They suggest that there is altered DLPFC functional connectivity with various cortical and subcortical regions during the resting state.^10^ DLPFC function is very important for maintaining executive memory cognition and working memory. DLPFC dysfunction affects incoming sensory information, language comprehension, reasoning and learning. Neurophysiological and neuroimaging studies have shown altered DLPFC functioning as one of the possible neural bases responsible for the cognitive deficits, such as poor episodic memory retrieval and executive function, noted in MCI patients.^11^ Anode placement over the left DLPFC and cathode over the right supraorbital region is the most common tDCS protocol for improving working memory.

There is a lack of effective treatments to prevent progression to dementia. Only a few studies have examined the efficacy of neuromodulation strategies for treatment of deficits associated to MCI or dementia. A single session of 1mA anodal tDCS improved word-retrieval of a group of 18 MCI patients in a study with a crossover design.^12^ Moreover, four sessions of 2mA anodal tDCS were also associated with cognitive improvement in mild vascular dementia.^13^ However, there are no studies about the effects of a longer protocol which might be suitable for current clinical practice, in terms of duration and weekly frequency.

## METHODS

### Participants

**Figure 1:**
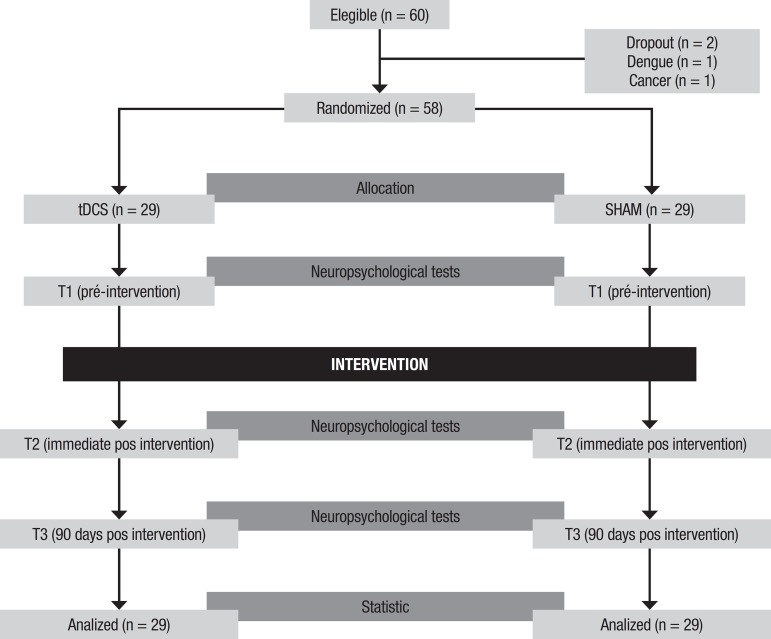
General study design.

Figure 1 shows the general study design. Sixty individuals aged over 60 years with MCI were recruited, of which 58 (20 males and 38 females) completed the study. Participants were assigned in order of spontaneous arrival at a medical clinic by a geriatric specialist, until a total of 60 participants was reached. The participants were then randomized into an active or sham group. The trial started after 60 individuals had been recruited, in order to achieve a 95% confidence interval with 12.75% confidence interval. Clinical diagnosis was based on the Mayo Clinic Criteria.^14^ Two individuals, one from each group, dropped out due to medical conditions unrelated to the study. Patients with unstable medical conditions, dementia and axis I psychiatric disorders, as well as subjects on psychotropic or anticholinergic drugs, were not included in the study.

### Ethics

The present study was approved by the UNIFESP ethics committee under number CAAE: 54213115.7.0000.5505. The study was not registered on clinical trials.

### Materials

Stimulation was delivered by a specialized device (brand Ibramed, model STRIAT GMES) with 25cm^2^ square rubber electrodes in a saline-soaked sponge. TDCS stimulation was administered by a trained biomedic, with no contact with the other evaluators. The instruments below were used for neuropsychological assessment. The Cambridge Cognitive Examination (CAMCOG) is a battery of psychological tests for cognitive assessment, comprised of several subscales to evaluate the following domains: orientation, language, memory, attention, praxis, perception, calculation and abstract thinking.^15^ The Mini-Mental State Examination test (MMSE) is a cognitive screening instrument assessing six dimensions: orientation, memory, attention, calculus, language and praxis.^16^ The Trail Making Test, comprising two versions, is a test which evaluates visual attention and task switching.^17^ The Semantic Verbal Fluency test (Animal word version) (SVF) evaluates verbal fluency by asking the individual to name as many different animals they can in one minute.^18^ The Boston naming test assesses verbal memory by presenting pictures of everyday objects and asking the subject to name them.^19^ The Clock Drawing Test entails a task where the individual is asked to draw a clock, used to assess visuospatial and praxis abilities.^20^ The Word List Memory Test (WLMT) comprises three phases, in which the individual is presented 10 words and has to recall them after 90 seconds and after 15 minutes from among 10 other distractors.^21^ The Digital Symbol-Coding test is a subtest from the Wechsler Adult Intelligence Scale which assesses processing speed, associative memory and graphomotor speed. The Forward and Backward Digit Span test is a subtest from the Wechsler Memory Scale which assesses verbal working memory and attention.^22^ The N-back test comprises a computer-test in which the individual is presented a sequence of stimuli, displayed one by one, and performs the task of matching the current stimulus with another presented n steps earlier in the sequence. We also applied the Hamilton Depression Rating Scale (HAM-D). These neuropsychological tests were administered by a blinded trained neuropsychologist who had no contact with the other evaluators.

### Procedures

We report the results of a randomized double-blind controlled trial of anodal tDCS assessing cognition. A current of 2.0 mA was applied for 30 minutes for 10 sessions, twice a week. The anode was placed over the left dorsolateral prefrontal cortex (LDLFC) and the cathode in the right supraorbital area. Sham stimulation involved the same set-up, but the current was turned off after a 30-second ramp. Figure 1 depicts the patient allocation and procedure protocol.

### Statistical analysis

Group comparisons were performed using the Mann-Whitney test and Pearson’s Chi square test. Differences between groups involving neuropsychological measures at baseline and after intervention were assessed with generalized estimating equations (GEE) (Gamma distribution and first-order autoregressive correlation matrix). Post-hoc pairwise comparison was corrected for multiple comparisons using least significant difference.

## RESULTS

**Figure 2:**
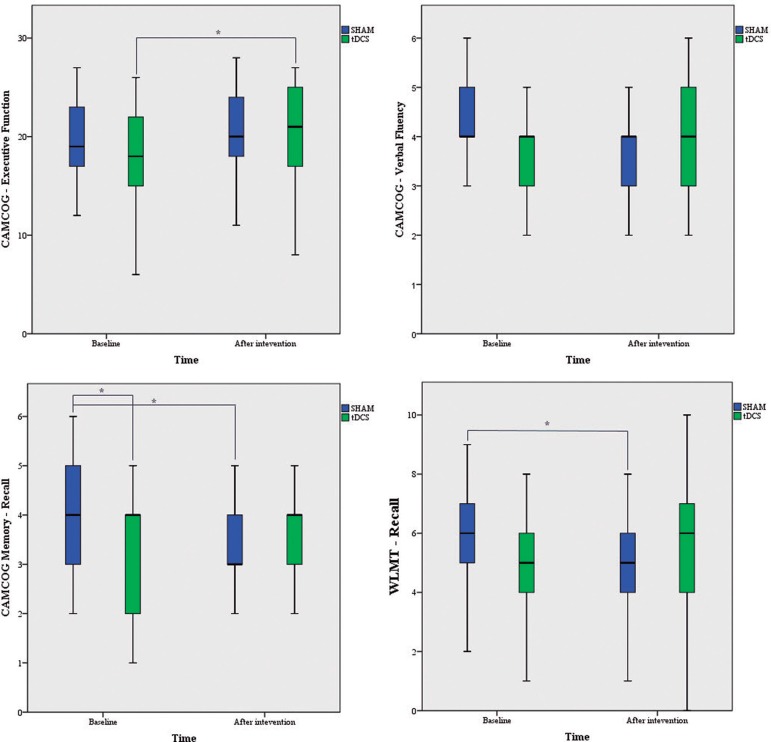
Boxplot showing results for both groups at pre and post intervention. *Significant differences (corrected with LSD).

Groups were matched for age (u(56) = 455; p = 0.591), gender (χ^2^(1) = 0.605; p = 0.581) and education level (χ^2^(2) = 4.971; p = 0.083). No significant differences were found in blood pressure, laboratory blood measures, cranial MRI aspects or HAM-D scores. Table 1 shows the clinical characteristics of the tDCS and Sham groups. Table 2 shows comparisons involving neuropsychological parameters between baseline and after 10 sessions of tDCS/Sham stimulation. After 10 sessions of tDCS, significant group-time interactions for the CAMCOG - executive functioning (χ^2^ = 3.961, p = 0.047), CAMCOG - verbal fluency (χ^2^ = 3.869, p = 0.049), CAMCOG Memory - recall (χ^2^ = 9.749, p = 0.004), and WMLT - recall (χ^2^ = 7.254, p = 0.007) were evident. A decline in performance for the CAMCOG - constructional praxis (χ^2^ = 4.371, p = 0.037) was found in the tDCS group after intervention. No significant effects involving the interaction between time and group were found for any other tasks. Figure 2 shows effects on neuropsychological parameters after tDCS × Sham interventions

**Table 1:** Summary of clinical characteristics and test results for group comparison.

	Active group (29)	SHAM group (29)	Sig.
Age in years (mean ± SD)	73.0 ± 9.2	71.6 ± 7.9	0.38
Sex – no. of women (%)	20 (69.0)	22 (75.9)	0.42
Systolic arterial pressure (mean ± SD)	127.3 ± 9.4	129.3 ± 7.7	0.35
Diastolic arterial pressure (mean ± SD)	79.2 ± 5.7	80.4 ± 4.6	0.54
Hemoglobin (mean ± SD)	13.0 ± 0.9	13.5 ± 0.7	0.35
Blood glucose (mean ± SD)	91.6 ± 11.4	89.7 ± 8.9	0.60
TSH (mean ± SD)	2.5 ± 2.1	5.0 ± 0.2	0.06
Sodium (mean ± SD)	140.7 ± 2.6	141.3 ± 2.7	0.44
Vitamin B12 (mean ± SD)	461.6 ± 216.5	527.3 ± 391.6	0.52
PCR (mean ± SD)	5.4 ± 5.7	2.88 ± 3.2	0.45
Cholesterol (mean ± SD)	190.4 ± 47.9	180.9 ± 36.2	0.82
HDL-C (mean ± SD)	55.8 ± 14.5	51.6 ± 3.5	0.15
Educational level	.	.	0.11
Middle school. n (%)	4 (13.8)	9 (31.1)	.
High school. n (%)	6 (20.6)	6 (20.6)	.
University. n (%)	19 (65.5)	14 (48.3)	.
Cranial MRI	.	.	0.08
RMC 0 n (%)	1 (3.4)	7 (24.1)	.
RMC 1 n (%)	23 (79.3)	19 (65.5)	.
RMC 2 n (%)	5 (17.3)	3 (10.4)	.
BDNF polymorphism	.	.	0.05
Genotype G/G	20 (69)	19 (65.5)	.
Genotype A/G	9 (31)	10 (34.5)	.

**Table 2:** Summary of cognitive test results comparing pre and post-intervention for each group, derived from repeated measures GEE.

Test	Group	Pre-intervention			Post-intervention		P_time_	P_group_	P_group time_
		Mean	Standard error		Mean	Standard error			
**CAMCOG**									
Executive functioning	SHAM	113.55	1487		116	1.271	0.709	0.001	0.047
	Active	111.17	2130		115.31	2.334			
Constructional praxis	SHAM	2.28	0.137		2.69	0.139	0.146	0.609	0.037
	Active	2.43	0.132		2.36	0.145			
Total language	SHAM	27.45	0.27		27.28	0.344	0.374	0.963	0.116
	Active	27.03	0.401		27.66	0.332			
Motor response	SHAM	3.86	0.064		3.86	0.064	0.315	0.632	0.315
	Active	3.83	0.098		3.97	0.034			
Verbal answer	SHAM	2.93	0.047		3	0.049	0.763	0.698	0.362
	Active	2.97	0.059		2.93	0.047			
Reading	SHAM	2	0		2.03	0.034	0.980	0.096	0.150
	Active	1.97	0.034		1.93	0.047			
Settings	SHAM	5.62	0.133		5.66	0.132	0.130	0.733	0.215
	Active	5.41	0.192		5.76	0.093			
Picture naming	SHAM	7.86	0.08		7.76	0.18	0.527	0.519	0.750
	Active	7.9	0.057		7.86	0.064			
Verbal fluency	SHAM	4.17	0.162		3.9	0.171	0.772	0.876	0.049
	Active	3.97	0.192		4.17	0.201			
Memory	SHAM	20.59	0.456		21.48	0.432	0.006	0.731	0.766
	Active	20.24	0.666		21.34	0.655			
Memory recall	SHAM	4	0.213		3.32	0.202	0.303	0.361	0.004
	Active	3.28	0.228		3.58	0.186			
Memory recognition	SHAM	5.21	0.165		5.41	0.134	0.409	0.394	0.560
	Active	5.14	0.181		5.17	0.169			
Remote Memory	SHAM	4.31	0.213		4.83	0.176	0.003	0.434	0.345
	Active	4.62	0.198		4.9	0.209			
Recent memory	SHAM	3.69	0.139		3.79	0.113	0.752	0.151	0.253
	Active	3.62	0.124		3.45	0.151			
Fixing address	SHAM	3.61	0.234		4.28	0.145	<0.001	0.696	0.881
	Active	3.72	0.219		4.34	0.164			
Heads up	SHAM	6.17	0.234		6	0.218	0.918	0.621	0.321
	Active	5.83	0.32		6.03	0.251			
Calculation	SHAM	1.85	0.067		1.83	0.07	0.478	0.939	0.300
	Active	1.79	0.075		1.9	0.077			
Praxis	SHAM	10.79	0.185		11.14	0.193	0.125	0.157	0.626
	Active	10.52	0.252		10.69	0.224			
Ideational praxis	SHAM	3.76	0.093		3.76	0.079	0.810	0.584	0.810
	Active	3.83	0.07		3.79	0.09			
Constructional praxis	SHAM	2.28	0.137		2.69	0.139	0.146	0.609	0.037
	Active	2.43	0.132		2.36	0.145			
Ideomotor praxis	SHAM	4.76	0.079		4.69	0.11	0.694	0.089	0.269
	Active	4.41	0.158		4.55	0.115			
Tactile perception	SHAM	2	0		1.97	0.034	0.565	0.556	0.565
	Active	1.97	0.034		1.97	0.034			
Visual sense	SHAM	7.52	0.143		6.93	0.188	<0.001	0.812	0.908
	Active	7.59	0.15		6.97	0.21			
Abstract thinking	SHAM	6.1	0.317		6.38	0.332	0.011	0.691	0.156
	Active	5.63	0.301		6.59	0.303			
Time orientation	SHAM	4.72	0.096		4.86	0.064	0.994	0.510	0.112
	Active	4.79	0.075		4.66	0.132			
Spatial orientation	SHAM	4.83	0.07		4.9	0.057	0.701	0.905	0.08
	Active	4.9	0.057		4.93	0.047			
Total	SHAM	113.55	1.487		116	1,271	<0.001	0.536	0.295
	Active	111.17	2.130		115.31	2,334			
Final	SHAM	93.93	0.979		95.83	0.686	0.001	0.579	0.742
	Active	92.83	1.447		95.14	1,602			
**Trail Making Test**									
Version A – time	SHAM	0.5697	0.06614		0.6462	0.08633	0.631	0.104	0.09
	Active	0.8269	0.11021		0.7707	0.09325			
Version A – errors	SHAM	1.13	0.117		1.12	0.122	0.780	0.623	0.765
	Active	1.25	0.23		1.29	0.352			
Version B – time	SHAM	24.872	0.29003		24,503	0.3288	0.874	0.962	0.929
	Active	24.559	0.25994		24,455	0.25501			
Version B – errors	SHAM	3	0.403		2.49	0.889	0.497	0.665	0.610
	Active	3	1,156		1.84	0.563			
**Word List Memory Task**									
WLMT-A1	SHAM	4.9	0.241		5.05	0.298	0.503	0.404	0.694
	Active	4.69	0.244		4.86	0.307			
WLMT-A2	SHAM	6.24	0.222		6.52	0.279	0.061	0.724	0.310
	Active	6.1	0.29		6.17	0.289			
WLMT-A3	SHAM	6.79	0.282		7.17	0.285	0.263	0.838	0.524
	Active	6.86	0.242		6.97	0.295			
WLMT-recall	SHAM	5.85	0.302		5.14	0.361	0.987	0.694	0.007
	Active	4.97	0.351		5.66	0.451			
WLMT-recall test: intrusions	SHAM	1.28	0.171		1.22	0.139	0.941	0.205	0.851
	Active	1.57	0.396		1.6	0.357			
WLMT-recognition test	SHAM	9.1	0.171		8.93	0.212	0.412	0.615	0.995
	Active	8.97	0.21		8.79	0.343			
WLMT-recognition test – intrusions	SHAM	1.28	0.176		0.96	0.103	0.395	0.021	0.091
	Active	1.65	0.284		1.81	0.363			
WLMT-total	SHAM	17.93	0.589		18.59	0.697	0.373	0.593	0.789
	Active	17.66	0.66		18	0.801			
**Other tests**									
Semantic Verbal Fluency test (Animal word version)	SHAM	17.31	0.867		16.86	0.903	0.81	0.874	0.268
	Active	16.55	0.972		17.24	0.971			
Mini-Mental State Examination test	SHAM	27.31	0.369		27.31	0.297	0.751	0.578	0.751
	Active	26.93	0.5		27.14	0.48			
Boston Naming test	SHAM	13.31	0.342		13.48	0.34	0.179	0.682	0.816
	Active	13.1	0.31		13.34	0.273			
Hamilton Depression Rating Scale	SHAM	8.66	1.003		6.74	0.712	0.033	0.117	0.459
	Active	10.31	1.394		9.13	1,259			
Clock Drawing Test	SHAM	8.759	0.2881		9,241	0.1883	0.012	0.401	0.743
	Active	8.345	0.4059		8,948	0.3957			
N-back	SHAM	508	0		508	0	0.312	0.312	0.312
	Active	500.48	7.386		508	0			
WAIS III - Code	SHAM	39.07	3.225		38.93	3.276	0.7	0.613	0.77
	Active	41.76	3.525		40.69	3.129			
WAIS III – Digit span DO	SHAM	7.38	0.403		7.45	0.435	0.785	0.734	0.959
	Active	7.52	0.337		7.62	0.385			
WAIS III – Digit span IO	SHAM	4.17	0.201		3.79	0.277	0.184	0.45	0.595
	Active	4.31	0.316		4.14	0.283			

## DISCUSSION

Our results suggest that tDCS can improve some aspects of memory impairment in elderly with MCI. We found significant changes in memory recall and long-term memory after administration of 10 sessions of tDCS twice a week.

Some authors have demonstrated advantages with the use of tDCS in treatment of mental disorders, particularly depression and cognitive impairment.^23^^,^ 24 Although most studies demonstrate that tDCS is a safe and effective method in depression and possibly Alzheimer disease,^25^ there are important issues to be considered. First, there is a lack of studies on tDCS efficacy for Mild Cognitive Impairment in the elderly. There are also doubts about the best techniques relating to the intensity of current, stimulation time, electrode placement and number and frequency of sessions. When studying actual results, we note variability of findings and conclusions, suggesting that numerous different factors may affect the results. Besides the variability of protocols, there is evidence in literature that genetic factors, such as Brain-Derived Neurotrophic Factor (BDNF) polymorphism, may influence the improvement in cognition after brain stimulation.^26^ We believe that a better understanding of neuroplasticity genes will be important to predict outcomes in tDCS.

Another important practical consideration is that trials usually involve daily sessions, which span a period of 4 weeks. This protocol is not affordable for most patients. In this sense, our premise in testing the efficacy of 30 min sessions, twice a week over 5 weeks was precisely to verify whether a more economical paradigm could also lead to positive results. Our results suggest that, using a current of 2 mA for 30 min twice a week over 5 consecutive weeks, tDCS is superior to placebo (Sham) for improvement of memory recall, verbal fluency and executive functioning in elderly with MCI. This study has some limitations: it was not possible to calculate the sample size because this was a pilot study. Nevertheless, the confidence interval was calculated for a sample of 60 individuals considering a 95% significance level and population of 209.3 million population. Although Fisher’s LSD was used, the statistical analysis did not employ more conservative methods for multiple comparison corrections such as Bonferroni or Sidak. The protocol was not registered in clinical trials, but was approved and followed by the Ethics Committee of the Unifesp (São Paulo Federal University). Despite other limitations of the study, including time and frequency of stimulation and number of subjects, results indicate a positive and promising therapeutic role for tDCS use in aging-related working memory dysfunction.

Further research involving larger trials and comparing different clinical protocols for this cohort is needed until translation to clinical practice can occur. More systematic research into this treatment alternative might help improve cognitive dysfunction in aging and related disorders.
